# Predicting Change in Posttraumatic Distress Through Change in Coping Self-Efficacy After Using the My Trauma Recovery eHealth Intervention: Laboratory Investigation

**DOI:** 10.2196/10309

**Published:** 2018-11-29

**Authors:** Charles C Benight, Kotaro Shoji, Carolyn M Yeager, Pamela Weisman, Terrance E Boult

**Affiliations:** 1 Department of Psychology University of Colorado Colorado Springs, CO United States; 2 Trauma, Health, and Hazards Center University of Colorado Colorado Springs, CO United States; 3 Department of Computer Science University of Colorado Colorado Springs, CO United States

**Keywords:** eHealth, posttraumatic stress disorder (PTSD), coping self-efficacy (CSE), trauma triggers, relaxation, digital behavior change interventions (DBCI), internet

## Abstract

**Background:**

Technology offers a unique platform for delivering trauma interventions (ie, eHealth) to support trauma-exposed populations. It is important to evaluate mechanisms of therapeutic change in reducing posttraumatic distress in eHealth for trauma survivors.

**Objective:**

This study evaluated a proactive, scalable, and individually responsive eHealth intervention for trauma survivors called My Trauma Recovery. My Trauma Recovery is an eHealth intervention aiming to support trauma survivors and consisting of 6 modules: relaxation, triggers, self-talk, professional help, unhelpful coping, and social support. It was designed to enhance trauma coping self-efficacy (CSE). We tested 3 hypotheses. First, My Trauma Recovery would decrease posttraumatic stress symptoms (PTSS). Second, My Trauma Recovery would increase CSE. And last, changes in CSE would be negatively correlated with changes in PTSS.

**Methods:**

A total of 92 individuals exposed to trauma (78/92, 85% females, mean age 34.80 years) participated. Our study was part of a larger investigation and consisted of 3 sessions 1 week apart. Participants completed the baseline online survey assessing PTSS and CSE. Each session included completing assigned modules followed by the online survey assessing CSE. PTSS was remeasured at the end of the last module.

**Results:**

PTSS significantly declined from T1 to T9 (*F*_1,90_=23.63, *P*<.001, η^2^_p_=.21) supporting the clinical utility of My Trauma Recovery. Significant increases in CSE for sessions 1 and 2 (*F*_8,83_=7.51, *P*<.001) were found. No significant change in CSE was found during session 3 (N=92). The residualized scores between PTSS T1 and T9 and between CSE T1 and T9 were calculated. The PTSS residualized score and the CSE residualized score were significantly correlated, *r*=–.26, *P*=.01. Results for each analysis with a probable PTSD subsample were consistent.

**Conclusions:**

The findings of our study show that participants working through My Trauma Recovery report clinically lower PTSS after 3 weeks. The results also demonstrate that CSE is an important self-appraisal factor that increased during sessions 1 and 2. These improvements are correlated with reductions in PTSS. Thus, changes in CSE may be an important mechanism for reductions in PTSS when working on a self-help trauma recovery website and may be an important target for eHealth interventions for trauma. These findings have important implications for trauma eHealth interventions.

## Introduction

### Mechanisms of Change for eHealth Interventions

eHealth interventions have demonstrated successful outcomes in reducing posttraumatic stress symptoms (PTSS) [1]. A meta-analytic study showed that eHealth interventions had medium to large effect sizes in reducing PTSS and trauma-related panic disorder, and the efficacy of the interventions was comparable to face-to-face therapy [2]. Amstadter and colleagues [1] argued that features such as psychoeducation, goal setting, exposure, and theoretical basis (eg, cognitive behavioral therapy) enhanced the efficacy of eHealth interventions in reducing symptoms. Most importantly, the more extensively eHealth interventions are developed based on a theory, the better their outcomes [2]. eHealth interventions that are based on theoretical models provide the opportunity to evaluate mechanisms of change that are predicted based on the theory. Understanding mechanisms of change through empirical experimental analysis provides important information for enhancing eHealth interventions. My Trauma Recovery (MTR) is a theoretically designed eHealth intervention for trauma survivors. The focus of this paper is to evaluate coping self-efficacy (CSE) as a key theoretically based mechanism of empowerment for users of MTR.

Benight and Bandura [3] suggested that social cognitive theory (SCT) provides a useful framework to understand trauma adaptation. Trauma recovery requires that individuals manage both extreme internal (eg, intrusive thoughts, hyperarousal) and external (eg, on-going posttraumatic stressors) demands putting a spotlight on self-regulation. SCT posits that self-regulation is managed through bidirectional interactions among environmental conditions, coping behaviors, and person factors [4]. Human beings use self-evaluation to determine success or failure in attaining valued goals (eg, regaining a sense of normalcy), thereby making coping adjustments based on environmental feedback. CSE perceptions are a primary factor in this self-evaluation feedback system predicting empowered perseverance or resigned giving up [4].

Previous studies have investigated effects of trauma and a posttrauma recovery process within the SCT framework. These studies examined the effects of trauma-specific CSE on PTSS. CSE appraisals were negatively associated with PTSS among survivors of natural disasters [5,6], terrorist attacks [7], motor vehicle accidents [8,9], and childhood sexual abuse [10]. In addition, CSE has shown to be a strong mediator between trauma-related distress variables (eg, negative cognitions) and negative outcomes [5,10]. It is important to note that a meta-analytic review of CSE in trauma adaption demonstrated effect sizes for longitudinal studies ranging from *r*=–.55 to *r*=–.62 with negative psychological outcomes [11]. These effect sizes are much stronger than other predictors often cited in posttraumatic outcome studies (eg, dissociation, *r*=.35; previous psychopathology, *r*=.17; or social support, *r*=–.28) [12]. Thus, CSE perceptions provide a useful target for evaluation as a mechanism of change that has shown to be significantly related to important posttraumatic outcomes. Our study examined the importance of CSE changes in psychological improvement while working through modules of the MTR website.

### My Trauma Recovery Description and Evaluation

**Figure 1:**
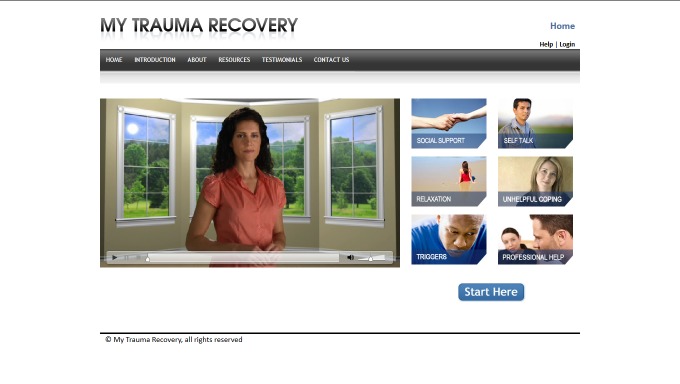
Homepage of the My Trauma Recovery website.

MTR offers 6 self-directed modules (relaxation, triggers, social support, professional help, self-talk, and unhelpful coping; see Figure 1 for the homepage of the website) based on SCT and underlying cognitive behavioral principles of self-management. The interactive website uses video and audio segments for modeling, feedback on progress to promote mastery, verbal persuasion through encouraging text, and physical arousal management through relaxation training [4]. Evidence for the clinical effectiveness of MTR is based on a study in the United States and a set of studies in China [13,14]. A randomized controlled trial (RCT) of the disaster recovery version of MTR showed that the website, compared to an information-only website, significantly reduced worry among survivors of Hurricane Ike (partial η^2^=.11, large effect size) [14]. In this study, CSE perceptions increased in the intervention group (η^2^=.05, medium effect size), although the effect was not statistically significant due to limited statistical power. An RCT of the Chinese version of MTR showed a significant reduction in PTSS after 1 month with a large effect size (Cohen *d*=0.81) and remained strong at the 3-month follow-up (Cohen *d*=0.87) in an urban sample. The effect was even stronger in a rural sample both at posttest (Cohen *d*=1.34) and at the 3-month follow-up (Cohen *d*=0.99) [13]. However, CSE did not significantly increase in this RCT. Thus, early studies suggest that MTR can help improve mental health following traumatic exposure. However, it remains unclear how important CSE perceptions are as a mechanism of change in PTSS when working with these eHealth interventions. This study provides evidence to help fill this void.

### This Study

This study analyzed changes in CSE perceptions throughout the sessions and how these changes related to changes in PTSS between pre- and post-sessions. The module order manipulation also allowed us to evaluate the importance of differential skill building in relation to changes in CSE and PTSS. Collectively, these data provide critical information to evaluate the importance of changes in CSE perceptions as a mechanism of change in the reduction in PTSS among trauma-exposed populations working on an eHealth intervention. This study was a part of a larger project to develop learning-based computer models mapping from sensory and facial/voice data to engagement, arousal, and self-efficacy states. The larger study used machine learning to develop a smart system to help maximize user benefit from the site.

### Hypotheses

The following hypotheses were generated for this study:

- Hypothesis 1: We hypothesized that PTSS would decrease from baseline to completion of all 3 sessions because previous studies have demonstrated a positive effect for trauma survivors using the site.
- Hypothesis 2: We hypothesized that CSE would increase as users engaged in the website because the site was designed based on SCT with specific interactive features to promote greater CSE (ie, personal empowerment).
- Hypothesis 3: We predicted that changes in CSE would be positively associated with reductions in PTSS.

## Methods

### Participants

#### Overview

A large diverse sample was purposively recruited in order to gather a wide range of responses to the MTR website to assist the machine learning aspect of the larger study. This also provides greater external validity for our study. Participants were survivors of domestic violence recruited from a local shelter, patients at local mental health clinics, people listed on a study registry at a trauma clinic, and undergraduate students who were enrolled in psychology courses at a university in the Mountain region of the United States. The inclusion criteria for this study were that participants must be aged 18 years or older and have had a traumatic experience within the past 2 years. In total, 93 trauma-exposed individuals completed session 1. One participant was excluded because that person worked on a different module from the one assigned, resulting in 92 individuals exposed to trauma (78/92, 85% females, mean age 34.80 years, SD 14.15) in session 1. Among these participants, 82 completed session 2, and 76 participated in all 3 sessions.

Table 1 shows demographic information of participants. Participants reported exposure to a wide range of traumatic events in the past 2 years including intimate partner abuse (35/92, 38%), sudden death of a close friend or loved one (27/92, 29%), threat of death or serious bodily harm (26/92, 28%), motor vehicle accidents (19/92, 21%), adult sexual abuse or assault (19/92, 21%), other accidents (14/92, 15%), severe assault by acquaintance or stranger (14/92, 15%), life-threatening illness (12/92, 13%), witness to family violence (12/92, 13%), miscarriage (12/92, 13%), natural disasters (9/92, 10%), witness to a severe assault of acquaintance or stranger (7/92, 8%), combat (5/92, 5%), life-threatening or permanently disabling illness of a loved one (5/92, 5%), childhood physical abuse (4/92, 4%), robbery involving a weapon (2/92, 2%), childhood sexual abuse by someone at least 5 years older (2/92, 2%), abortion (1/92, 1%), and other (6/92, 7%).

#### Posttraumatic Stress Symptoms

Posttraumatic stress disorder (PTSD) Checklist Version 5 (PCL-5) was used to assess 4 symptom clusters of PTSD (intrusion, avoidance, negative alterations in cognitions and mood, and alterations in arousal and reactivity) corresponding to the *Diagnostic and Statistical Manual of Mental Disorders, Fifth Edition*, symptom criteria [15,16]. The PCL-5 is a 20-item measure assessing how bothersome each symptom was in the past month on a 5-point scale ranging from 0 (not at all) to 4 (extremely). Respondents were asked to complete each item with the stem “In the past month, how much were you bothered by...” Sample items included “Repeated, disturbing, and unwanted memories of the stressful experience” and “Trouble remembering important parts of the stressful experience.” Scores range between 0 and 80. Cronbach alpha coefficients were .95 at the baseline and .96 at the end of session 3.

#### Trauma Coping Self-Efficacy

The Trauma Coping Self-Efficacy Scale was used to assess coping self-efficacy appraisals for dealing with posttrauma challenges [17]. The scale comprises 9 items assessing the perception of capability to deal with internal and external demands on a 7-point scale ranging from 1 (not at all capable) to 7 (totally capable). Respondents answered each question with the stem “I am capable to...” Sample items included “Get my life back to normal” and “Not ‘lose it’ emotionally.” Total scores range between 1 and 49 and overall mean scores from 1 to 7. Overall mean scores are offered for ease of interpretation. Internal reliability coefficients were .89 at the baseline (T1), .92 after module 1 in session 1 (T2), .93 after module 2 in session 1 (T3), .92 at the beginning of session 2 (T4), .92 after module 1 in session 2 (T5), .93 after module 2 in session 2 (T6), .92 at the beginning of session 3 (T7), .93 after module 1 in session 3 (T8), and .95 after module 2 in session 3 (T9).

#### Trauma History

Traumatic Life Events Questionnaire was used to assess whether respondents have had a traumatic experience in the past 2 years [18]. It is a list of 22 traumatic events where respondents answer with “yes” or “no” depending on whether or not they have experienced the event. Sample items included natural disasters, intimate partner abuse, and robbery involving a weapon.

### Procedures

**Figure 2:**
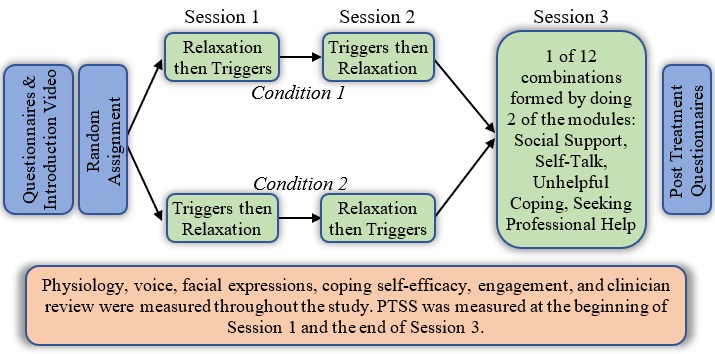
Flowchart of the study procedures. PTSS: posttraumatic stress symptoms.

Qualified participants were invited to the laboratory for 3 sessions, each 1 week apart (see Figure 2 for the study design). Upon arrival to the lab, participants were asked by a research assistant to wash their hands to ensure accurate measurement of skin conductance. Participants then completed the baseline online survey assessing PTSS, CSE, and demographics. After participant completion of the baseline online survey (T1), a research assistant attached the respiration band and electrodes for the electrocardiogram and skin conductance.

Participants watched an introductory video that described an overview of the website. Immediately following the video, participants were asked to close their eyes and relax for 1 minute in order to gather baseline physiological assessments. Next, participants worked on the randomly assigned first module (either triggers or relaxation) followed by the online survey assessing CSE (T2). This procedure was repeated for the second module (T3). One week later participants repeated the same process except they completed the CSE before they began the module (T4). The order of the modules was counterbalanced as they completed the 2 modules again. CSE was assessed after each module (T5 and T6). The final session included the same procedures used in sessions 1 and 2, except for the modules participants completed. Two modules were randomly selected from the remaining 4 modules (ie, seeking professional help, social support, unhelpful coping, self-talk). CSE was again assessed before they worked on the modules (T7) and after they finished each module (T8, T9). The participants also completed the PCL-5 at the end of the last module. Participants received US $25 after each session. Local and national mental health resources were provided to all participants after the study.

### Statistical Analysis

First, we performed a 2 (module order) × 2 (assessment period) mixed multivariate analysis of variance (MANOVA) on PTSS to test whether PTSS improved after the use of MTR (hypothesis 1). Dependent variables were PTSS at baseline and the end of the study. Significant effects were followed up with post hoc follow-up tests using the Fisher least significant difference method.

Similarly, we conducted a 2 (module order) × 3 (session) × 3 (assessment period) MANOVA on CSE across all time points to analyze whether CSE increased as participants continued to use the website (hypothesis 2). SPSS Statistics version 24 (IBM Corp) was used for the analysis. Dependent variables included CSE at baseline, after module 1, and after module 2 for all 3 sessions.

The effect of change in CSE on changes in PTSS was evaluated using a bivariate correlation between the residualized change score for CSE from T1 to T9 and the residualized change score for PTSS T1 to T9.

### Missing Data Treatment

Missing data were imputed using the maximum likelihood estimation in analysis of a moment structures. The assumption of the maximum likelihood imputation is that missing data must be at least missing at random. Because there is no procedure to assess missing at random, we performed a test of Little missing completely at random, which is a stricter assumption than missing at random. Results of the Little missing completely at random test with the module order condition and relationship status as references showed that missing data were missing completely at random for all study variable items (χ^2^_453_=442.98, *P*=.62). Thus, all missing data were imputed. In total, 1.85% of the session 1 values, 11.27% of the session 2 values, and 17.41% of the session 3 values were imputed.

**Table 1:** Descriptive statistics for demographics (some percentages do not add up to 100% due to missing data; N=92).

Variable		Value
Age (years) mean (SD)		14.15 (34.80)
Age (years), range		18-79
**Gender, n (%)**		
	Female	78 (84.8)
	Male	14 (15.2)
**Ethnicity, n (%)**		
	White	68 (73.9)
	African American	14 (15.2)
	Hispanic/Latino	9 (9.8)
	Native American/Alaskan	7 (7.6)
	Asian/Pacific Islander	5 (5.4)
	Other/prefer not to answer	2 (2.2)
**Intimate relationship, n (%)**		
	Single	32 (34.8)
	Divorced	21 (22.8)
	Married	17 (18.5)
	Separated	13 (14.1)
	Widowed	1 (1.1)
	Other	6 (6.5)
**Highest education, n (%)**		
	High school	24 (26.1)
	Some college	36 (39.1)
	Associates degree	15 (16.3)
	Bachelor’s degree	4 (4.3)
	Master’s degree	7 (7.6)
	Other	4 (4.3)
**Income (USD), n (%)**		
	$0-$25,000	52 (56.5)
	$25,001-$70,000	21 (22.8)
	$70,001-$100,000	2 (2.2)
	>$100,000	10 (10.9)
**Seeing mental health provider, n (%)**		
	Yes	50 (54.3)
	No	40 (43.5)

## Results

### Descriptive Data

Attrition analysis revealed there were no significant differences between session 1 and session 2 and session 2 and session 3 in sex, age, education, and baseline PTSS and CSE. There were no significant differences in baseline PTSS (*t*_90_=1.94, *P*=.06) or CSE (*t*_90_=1.46, *P*=.18) between the 2 module order conditions. Multimedia Appendix 1 displays bivariate correlation coefficients, means, and standard deviations for the study variables. Out of 92 participants, 54 (59%) reported PTSD scores greater than or equal to 33, which is considered a probable diagnostic level. Overall, CSE levels showed negative correlations with PTSS across study time points.

### Hypothesis 1: Posttraumatic Stress Symptoms Would Decrease From Baseline to Completion of All 3 Sessions

**Figure 3:**
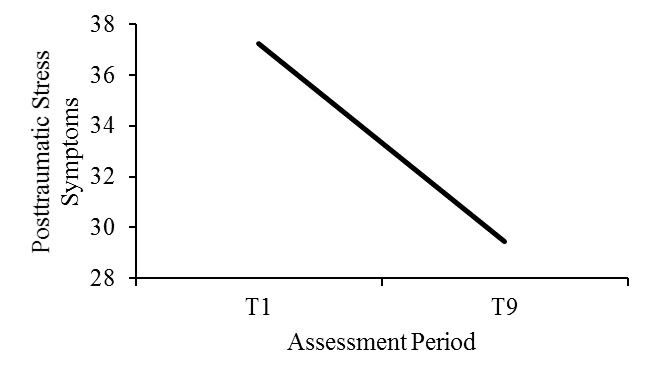
Change in posttraumatic stress symptoms from T1 to T9. T1: baseline, T9: after module 2 in session 3.

Findings of a 2 (module order) × 2 (assessment periods) MANOVA on PTSS showed that assessment periods were significant, indicating PTSS significantly declined over time (*F*_1,90_=23.63, *P*<.001, η^2^_p_=.21; see Figure 3). Participants’ T1 PTSS scores (mean 38.01, SD 19.56) suggested they had on average relatively high initial symptoms of PTSD. Scores above 33 are recommended for diagnosable PTSD [19]. The drop in average PTSS scores at T9 (mean 29.89, SD 17.17) was close to 10 points suggesting a clinically significant reduction of PTSD symptoms according to Weathers et al [19]. The main effect of the module order (*F*_1,90_=3.10, *P*=.08, η^2^_p_=.03) and the interaction between the module order and the assessment periods (*F*_1,90_=1.16, *P*=.29, η^2^_p_=.01) were not significant.

Results from a 2 (module order) × 2 (assessment period) MANOVA with participants with probable PTSD diagnosis showed consistent, yet stronger, results. The assessment period was significant (*F*_1,52_=39.07, *P*<.001, η^2^_p_=.43) demonstrating that levels of PTSS declined across the study. The module order (*F*_1,52_=0.13, *P*=.73, η^2^_p_=.002) and the interaction effect between the assessment period and the module order (*F*_1,90_=23.63, *P*<.001, η^2^_p_=.21) were not significant.

### Hypothesis 2: Trauma Coping Self-Efficacy Will Increase as Users Engage in the Website

**Figure 4:**
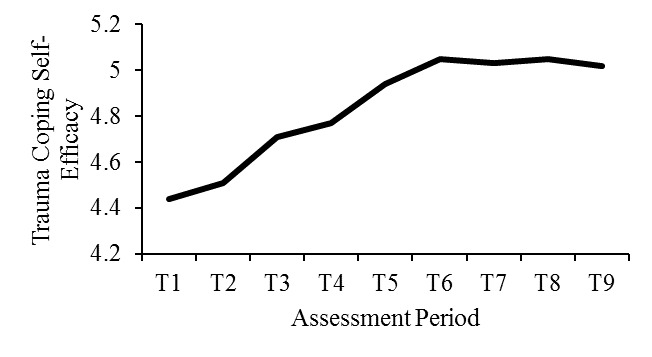
Change in trauma coping self-efficacy across assessment periods. T1: baseline, T2: after module 1 in session 1, T3: after module 2 in session 1, T4: at the beginning of session 2, T5: after module 1 in session 2, T6: after module 2 in session 2, T7: at the beginning of session 3, T8: after module 1 in session 3, T9: after module 2 in session 3.

Results of a 2 (module order) × 9 (assessment periods) mixed model MANOVA on CSE showed that CSE levels significantly improved across study time points (*F*_8,83_=7.51, *P*<.001, η^2^_p_=.42; see Figure 4), supporting hypothesis 2. Results of the follow-up tests with the least significant difference showed that from T1 to T3 (session 1; *t*_91_=3.30, *P*<.001), T4 to T6 (session 2; *t*_91_=4.31, *P*<.001), and T1 to T9 (all sessions; *t*_91_=3.70, *P*<.001), there were significant increases in CSE on average. There was no significant change in CSE from T7 to T9 (session 3; *t*_91_=0.41, *P*=.68). CSE did not significantly change from T3 to T4 (*t*_91_=0.38, *P*=.70) and from T6 to T7 (*t*_91_=0.03, *P*=.97). These results of the follow-up tests indicated that CSE significantly increased within sessions 1 and 2 but not within session 3 and between sessions. There was no significant effect of the module order (*F*_1,90_=0.81, *P*=.37, η^2^_p_=.01), and no significant interaction effect between the module order and the assessment periods (*F*_8,83_=1.96, *P*=.06, η^2^_p_=.16).

The 2 (module order) × 2 (assessment period) MANOVA on CSE with participants with probable PTSD showed consistent results (N=54). The assessment period was significant (*F*_8,45_=4.13, *P*<.001, η^2^_p_=.42, Cohen *d*=1.70). Follow-up tests showed that CSE was higher at T3 than T1 (*t*_53_=2.05, *P*=.045) and at T6 than T4 (*t*_53_=2.02, *P*=.049). There was no significant difference between T3 and T4 (*t*_53_=0.46, *P*=.65), T6 and T7 (*t*_53_=0.01, *P*=.99), or T7 and T9 (*t*_53_=1.27, *P*=.21). The module order was not significant (*F*_1,52_=1.86, *P*=.18, η^2^_p_=.04), and the interaction effect of the module order and the assessment period was not significant (*F*_8,45_=0.76, *P*=.63, η^2^_p_=.12).

### Hypothesis 3: Overall Improvement in Trauma Coping Self-Efficacy Would Predict Decreases in Posttraumatic Stress Symptoms

The bivariate correlations between the overall change in CSE and change in PTSS (using residualized change scores for both variables) demonstrated a significant negative correlation (*r*=–.26, *P=*.01), explaining approximately 7% of the variance. Among participants with probable PTSD, the correlation between the overall change in CSE and change in PTSS was slightly stronger and also significant (*r*=–.32, *P*=.02), accounting for 10% of the variance.

## Discussion

### Principal Findings

Our study provides important information on the clinical utility of MTR in reducing PTSS and the importance of changes in CSE perceptions as a mechanism of change in the reduction in PTSS. Results were supportive of study hypotheses. PTSS significantly decreased from baseline to the end of session 3 (η^2^_p_=.21, Cohen *d*=.41) suggesting a moderate effect size and supporting hypothesis 1. The effect was stronger for individuals with probable PTSD (η^2^_p_=.43, Cohen *d*=1.66). This result is consistent with a therapist-assisted cognitive behavior therapy internet intervention for a variety of trauma survivors (η^2^_p_=.27) and with an internet intervention for Iraqi people exposed to war (Cohen *d*=1.57) [20,21]. It should be noted that the Web-based intervention used in Iraq includes therapist assistance and exposure-based methods that MTR does not employ.

Notably, self-appraisals of coping capability to manage trauma recovery (CSE) increased across the first 2 study sessions. This implies that trauma survivors working on a self-help trauma recovery website may get the biggest boost to their confidence early when they initially use the relaxation and triggers management modules (hypothesis 2). Significant increases in CSE were also observed during both session 1 and session 2 for the overall sample and the clinical subsample. This is important in that the survivors were working through the same modules they had seen the week before. Thus, the initial boost in self-efficacy was enhanced with further exposure to skills designed to manage hyperarousal and triggering environmental stimuli.

CSE levels did not significantly change during session 3. The lack of continued improvement in self-efficacy perceptions from session 3 is difficult to interpret. It is possible that the first 2 sessions provided the maximum benefit for efficacy change. Indeed, CSE was close to maximum values by the end of session 2. The random assignment of the remaining 4 modules restricted our ability to tease apart module effects. These modules, seeking social support, self-talk, unhelpful coping, and seeking professional help, have differential levels of skill development that may specifically target CSE. Gaining skill in enhancing one’s social network and level of recovery support should, theoretically, promote self-efficacy beliefs (ie, enabling effect) [22]. In addition, management of negative self-talk by gaining the skill of positive reframing and dysfunctional thought identification is a standard in trauma treatment [23] and should also promote greater self-efficacy. With participants learning to be their own best advocate in the recovery process, CSE should improve relative to improved coping behaviors and lower distress. The remaining 2 modules may ultimately enhance CSE perceptions through contact with a professional helper and the reduction of negative coping behaviors (eg, anger, drug/alcohol use) but might take longer to generate positive effects. These speculations require further examination through sophisticated laboratory studies. The number of permutations of module order with 6 modules combined with session order requires an extremely large sample. Future studies with the MTR website that target the remaining 4 modules (offering them first) are necessary to more thoroughly evaluate their effectiveness in promoting survivor empowerment. This study is already underway.

Furthermore, we did not find a significant CSE change between sessions. Participants waited at least 1 week between sessions, during which they were not required to complete homework. Homework is usually an important part of a trauma treatment [24]. The finding that there was no increase in CSE between sessions might indicate the importance of homework assignments between sessions for a trauma treatment [25,26]. However, participants maintained the same levels of CSE between sessions even without homework assignments. Because CSE increased within sessions, the website might be helpful as a homework assignment between therapy sessions, a speculation that awaits future investigation.

Hypothesis 3 was supported by our findings. Change in CSE was significantly and negatively correlated with changes in PTSS in the full sample and the probable PTSD subsample. This suggests CSE is an important process variable relative to improvement in PTSS through an eHealth intervention system. MTR was designed based on SCT principles including mastery skill building, modeling, verbal persuasion, and arousal reduction to promote greater CSE beliefs. This finding suggests that those who improve their CSE also experience reductions in PTSS. The opposite is also evident, that reductions in symptoms undoubtedly drive up CSE. This bidirectional influence is consistent with SCT [4]. Importantly, our finding that changes in CSE appear to be early in use of the website (session 1 and session 2) suggests CSE might be a specific target for PTSS technology interventions. We did not measure changes in PTSS session by session, making it impossible to evaluate the correlations between CSE session changes and PTSS session improvements. Future studies that include these shifts may provide a clearer picture of the dynamics between self-appraisal changes and symptom reduction. In addition, RCTs are required to provide further evidence for MTR’s effectiveness in reducing PTSS and enhancing CSE by comparing changes in these variables in a treatment group and an adequate comparison group.

In a study on the MTR, Wang et al [13] did find reductions in PTSS compared to a control group. They also reported results on CSE change compared with a wait-list group after working on the Chinese version of the MTR. They found that CSE was not significantly different between the treatment and comparison groups after 1 month even though CSE slightly increased from the baseline to 1-month follow-up in the treatment group. In comparison, Steinmetz et al [14] did find increases in CSE following use of the My Disaster Recovery site (a sister site to MTR) after Hurricane Ike demonstrating a moderate effect size, although it should be noted that this effect was not statistically significant due to the low statistical power for this study. These inconsistencies between our study and the study conducted by Wang et al [13] might be due to cultural differences. An RCT of the English version of the MTR needs to be conducted to directly compare changes in CSE with the Chinese version.

### Clinical Implications

The first clinical implication is the possible value of the MTR website for reducing PTSS. As a standalone website, this provides a useful tool to assist survivors in their recovery. The CSE findings also have important implications for clinical interventions. Coping self-efficacy often serves a crucial role in other clinical interventions. For example, a group intervention for veterans with PTSD demonstrated increases in self-efficacy as a therapeutic target [27]. Wiedenfeld et al [28], in an experiment with spider phobics, showed that enhancing self-efficacy directly related to improvements in immune functioning. Therapeutic improvements in coping self-efficacy following a group intervention for HIV-positive patients mediated reductions seen in stress and burnout [29]. In another study with patients who contracted HIV, high CSE was related to adherence to antiretroviral treatment [30,31]. Last, changes in self-efficacy perceptions as well as baseline levels predicted both physical and psychological improvements in a sample of patients with multiple sclerosis [32]. Although these medical disorders are different than coping with trauma, these findings suggest that strong perceptions of capability to manage challenging demands are important in predicting important behavioral outcomes. Indeed, significant research with trauma survivors demonstrates the importance of CSE perceptions in healthy and unhealthy adaptation [3,11].

### Limitations

Several limitations should be considered when interpreting the findings in this study. First, the research design limits the causal interpretation of our data. A nontreatment control condition was not included, making the changes in PTSS or CSE difficult to interpret relative to the website versus general improvement over time. An RCT with an appropriate control condition is needed to more effectively evaluate the effect of MTR on PTSS and CSE. Second, we only examined the order effect for 2 of the 6 modules. The influence of order with the rest of the 4 modules was not investigated. Future studies must evaluate the order effects of the remaining 4 modules. Last, the use of the modules was in a controlled laboratory environment, which may have influenced our findings due to demand effects, interactions with laboratory personnel, and payment for participating versus a more natural use of the website.

### Conclusions

Our study examined whether using MTR resulted in significant reductions in PTSS, whether levels of CSE changed throughout the 3 study sessions over 3 weeks, and whether the changes in CSE predicted changes in PTSS after using the website. The findings confirmed that PTSS symptoms went down using MTR over the study period, trauma survivor CSE improved, and change in CSE correlated with changes in posttraumatic distress. Our study offers support for the clinical value of the standalone MTR and offers initial evidence for a therapeutic mechanism for the reduction in PTSS after working on a trauma recovery website.

## Appendix

Multimedia Appendix 1 Correlations, means, and standard deviations of posttraumatic stress symptoms and coping self-efficacy.

## Abbreviations

CSE: coping self-efficacy
MANOVA: multivariate analysis of variance
MTR: My Trauma Recovery
PCL-5: Posttraumatic Stress Disorder Checklist version 5
PTSD: posttraumatic stress disorder
PTSS: posttraumatic stress symptoms
RCT: randomized controlled trial
SCT: social cognitive theory
