# Supplementary material for: Predicting Change in Posttraumatic Distress Through Change in Coping Self-Efficacy After Using the My Trauma Recovery eHealth Intervention: Laboratory Investigation
Source: JMIR Ment Health. 2018 Nov 29;5(4):e10309. doi: 10.2196/10309 (PMC6293247; doi:10.2196/10309)
Supplement: Multimedia Appendix 1 [file mental_v5i4e10309_app1.pdf]

Multimedia Appendix 1. Correlations, means and standard deviations of PTSS and CSE-T.

Variables	1	2	3	4	5	6	7	8	9	10	11
1. T1 PTSS	--	.47[a]	-.46[a]	-.44[a]	-.41[b]	-.18	-.17	-.21	-.24	-.19	-.12
2. T9 PTSS	.67[a]	--	-.15	-.22	-.16	-.08	-.12	-.15	-.45[a]	-.39[b]	-.30
3. T1 CSE-T	-.61[a]	-.39[a]	--	.75[a]	.72[a]	.57[a]	.59[a]	.47[a]	.42[b]	.36[b]	-.22
4. T2 CSE-T	-.59[a]	-.44[a]	.87[a]	--	.90[a]	.51[a]	.54[a]	.52[a]	.42[b]	.35[b]	.37[b]
5. T3 CSE-T	-.57[a]	-.38[a]	.83[a]	.91[a]	--	.60[a]	.60[a]	.65[a]	.45[a]	.38[b]	.38[b]
6. T4 CSE-T	-.50[a]	-.39[a]	.62[a]	.61[a]	.62[a]	--	.94[a]	.81[a]	.60[a]	.63[a]	.59[a]
7. T5 CSE-T	-.49[a]	-.41[a]	.63[a]	.62[a]	.62[a]	.96[a]	--	.84[a]	.60[a]	.66[a]	.60[a]
8. T6 CSE-T	-.50[a]	-.44[a]	.57[a]	.61[a]	.65[a]	.87[a]	.91[a]	--	.66[a]	.70[a]	.67[a]
9. T7 CSE-T	-.56[a]	-.60[a]	.56[a]	.60[a]	.55[a]	.72[a]	.72[a]	.74[a]	--	.87[a]	.74[a]
10. T8 CSE-T	-.43[a]	-.47[a]	.45[a]	.45[a]	.43[a]	.60[a]	.62[a]	.64[a]	.81[a]	--	.86[a]
11. T9 CSE-T	-.33[a]	-.38[a]	.35[a]	.43[a]	.39[a]	.59[a]	.60[a]	.62[a]	.72[a]	.91[a]	--
$M_{\text{Full Sample}}$	38.01	29.90	4.44	4.51	4.71	4.77	4.94	5.05	5.03	5.05	5.02
$SD_{\text{Full Sample}}$	19.56	17.17	1.18	1.28	1.30	1.19	1.18	1.20	1.07	1.13	1.24
$M_{\text{Subsample}}$	51.77	38.04	3.93	3.96	4.19	4.30	4.46	4.55	4.54	4.67	4.69
$SD_{\text{Subsample}}$	12.06	15.79	0.93	1.06	1.15	1.01	1.02	1.12	0.98	0.96	1.09

Note. Correlations over dash (--) correspond to values for participants with PCL-5 scores ≥ 33 (probably diagnosable PTSD; *N* = 92); Correlations under dash correspond to values for all participants (*N* = 54). Full sample consisted of all participants; Subsample consisted of participants with PCL-5 scores ≥ 33. N = sample size; M = mean; SD = standard deviation; PTSS = total posttraumatic stress symptoms; CSE-T = trauma coping self-efficacy mean scores; T1 = baseline; T2 = Session 1 after the first module; T3 = Session 1 after the second module; T4 = Session 2 start; T5 = Session 2 after first module; T6 = Session 2 after the second module; T7 = Session 3 start; T8 = Session 3 after the first module; T9 = Session 3 after the second module. [a] $P < .001$, [b] $P < .01$, [c] $P < .05$.
